# Improved Pharmacokinetic Feasibilities of Mirabegron-1,2-Ethanedisulfonic Acid, Mirabegron-1,5-Naphthalenedisulfonic Acid, and Mirabegron-L-Pyroglutamic Acid as Co-Amorphous Dispersions in Rats and Mice

**DOI:** 10.3390/pharmaceutics15092277

**Published:** 2023-09-04

**Authors:** Seo-Yeon Kim, Byung Hoon You, Mingoo Bae, Seung Yon Han, Kiwon Jung, Young Hee Choi

**Affiliations:** 1 College of Pharmacy and Integrated Research Institute for Drug Development, Dongguk University_Seoul, 32 Dongguk-ro, Ilsandong-gu, Goyang-si 10326, Gyeonggi-do, Republic of Korea; kimsy7856@naver.com (S.-Y.K.); hoon4131@nate.com (B.H.Y.); nophra88@naver.com (M.B.); hsyglory@gmail.com (S.Y.H.); 2 College of Pharmacy, CHA University, 335 Pangyo-ro, Bundang-gu, Seongnam-si 13488, Gyeonggi-do, Republic of Korea; 3 Oncobix Co., Ltd., 120 Heungdeokjungang-ro, Giheung-gu, Yongin-si 16950, Gyeonggi-do, Republic of Korea

**Keywords:** mirabegron, co-amorphous dispersion, pharmacokinetics, bioavailability, tissue distribution

## Abstract

Mirabegron (MBR) is a β_3_-adrenoceptor agonist used for treating overactive bladder syndrome. Due to its poor solubility and low bioavailability (*F*), the development of novel MBR formulations has garnered increasing attention. Recently, co-amorphous dispersions of MBR, such as MBR-1,2-ethanedisulfonic acid (MBR-EFA), MBR-1,5-naphthalenedisulfonic acid (MBR-NDA), and MBR-L-pyroglutamic acid (MBR-PG), have been developed, showing improved solubility and thermodynamic stability. Nevertheless, the pharmacokinetic feasibility of these co-amorphous dispersions has not been evaluated. Therefore, this study aimed to characterize the pharmacokinetic profiles of MBR-EFA, MBR-NDA, and MBR-PG in rats and mice. Our results exhibited that relative *F*_24h_ and AUC_0–24h_ values of MBR in MBR-EFA, MBR-NDA, and MBR-PG rats were increased by 143–195% compared with the MBR rats. The absolute *F*_24h_, relative *F*_24h_, and AUC_0–24h_ values of MBR in MBR-EFA and MBR-NDA mice were enhanced by 178–234% compared with the MBR mice. In tissue distribution, MBR was extensively distributed in the gastrointestinal tract, liver, kidneys, lung, and heart of mice. Notably, MBR distribution in the liver, kidneys, and lung was considerably high in MBR-EFA, MBR-NDA, or MBR-PG mice compared with MBR mice. These findings highlight the potential of these co-amorphous dispersions to enhance oral *F* of MBR.

## 1. Introduction

Mirabegron (MBR), a selective β_3_-adrenergic receptor agonist, was developed for the treatment of overactive bladders [1,2]. In its active pharmaceutical ingredient (API) form, MBR belongs to BCS class III drugs [3,4]. MBR in a crystalline solid state exhibits a poor aqueous solubility of 0.082 mg/mL [5] and low bioavailability (*F*) [6,7,8] in addition to low permeability [9].

Although a crystalline form of MBR is currently marketed as Betmiga^®^ by Astellas Pharma [4,10], the challenge of addressing its solubility and its physicochemical stability issues persist [4]. Currently, the slow-release tablet of MBR used for overactive bladder syndrome patients utilizes the crystalline form [3]. Considering the significant impact of the solubility of an active ingredient on its bioavailability, the physical stability and solubility of MBR in the developed formulations are crucial [3]. Consequently, various polymer-based amorphous formulation of MBR have been employed to overcome its poor aqueous solubility and low *F* [6,7,11,12,13,14].

Amorphous drug formulations are known to enhance thermodynamic energy, solubility, and dissolution rates compared with crystalline solid forms. However, a drawback of amorphous drug formulation is the rapid conversion to a less soluble crystalline form due to weak thermodynamic stability. To prevent crystallization and maintain their amorphous state, co-formers have been used because of their hygroscopic properties, which promote thermodynamic stability and help retain the amorphous state of drugs [6,11,12,13,14,15,16,17]. As an appropriate co-former to form co-amorphous dispersion, the solubility and pKa values of co-formers are considered [6]. Also, the high glass transition temperature (T_g_) values of co-amorphous dispersions promote the storage stability of amorphous states during a long period by inhibiting the conversion of amorphous dispersions into crystalline solids [18,19,20,21,22]. Thus, as an appropriate co-former to form co-amorphous dispersions, the following chemical properties of co-formers are recommended: co-formers are water-soluble and the pKa difference between co-former and the drug is >3 [6,23]. An et al. [6] designed various co-amorphous dispersions of MBR, such as MBR-1,2-ethanedisulfonic acid (MBR-EFA), MBR-1,5-naphthalenedisulfonic acid (MBR-NDA), and MBR-L-pyroglutamic acid (MBR-PG). Importantly, it was proven that these co-amorphous dispersions improve the phase stabilities of MBR-EFA, MBR-NDA, and MBR-PG by promoting thermodynamic stability and preventing recrystallization. Consequently, the solubility of MBR increased in MBR-EFA, MBR-NDA, and MBR-PG [6]. However, the potential impact of the co-amorphous dispersions of MBR on the in vivo pharmacokinetic feasibility of MBR has not been evaluated yet. Therefore, the objective of this study is to characterize the absorption, distribution, and elimination properties, resulting in in vivo *F* values of MBR after the oral administration of co-amorphous dispersions of MBR in rats and mice. Additionally, the tissue distribution of MBR—encompassing the heart, lung, stomach, small intestine, large intestine, liver, kidneys, brain, and fat—was investigated after the oral administration of MBR and its co-amorphous dispersions in mice. The selection of these tissues was guided by their involvement in absorption through the digestive system, elimination through the liver and kidneys, (pre-)systemic circulation via the lung and heart, and susceptibility to the brain and fat due to the interplay of physicochemical similarities between the drug and tissues.

## 2. Materials and Methods

### 2.1. Chemicals

**Figure 1:**
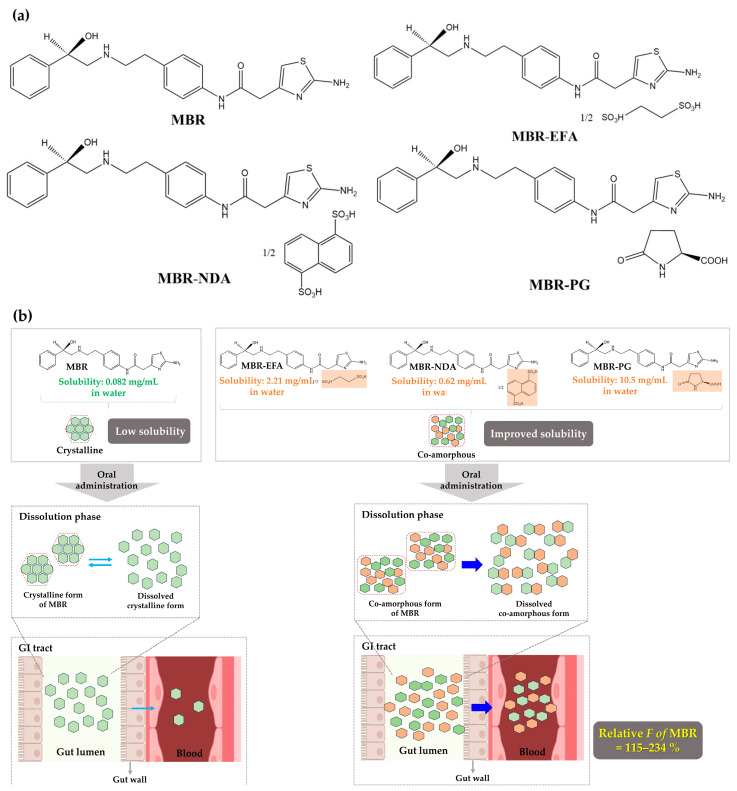
(**a**) Chemical structures of MBR, MBR-EFA, MBR-NDA, and MBR-PG. (**b**) Absorption process after oral administration of MBR and its co-amorphous dispersions.

MBR, MBR-EFA, MBR-NDA, and MBR-PG (Figure 1a) were supplied by Dr. Kiwon Jung (CHA University, Seongnam-si, Republic of Korea and Oncobix Co., Ltd., Yongin-si, Republic of Korea) [6,24]. Finasteride [internal standard (IS) of ultra-performance liquid chromatography-tandem mass spectrometry (UPLC-MS/MS)] was purchased from Sigma-Aldrich (St. Louis, MO, USA). All other chemicals used in the study were of analytical grade.

### 2.2. Animals

The animal study protocols were approved by the Institute of Laboratory Animal Resources of Dongguk University_Seoul, Republic of Korea (Approval no. IACUC-2019-025, 2019; Approval no. IACUC-2022-012, 2022). Male Sprague–Dawley rats (6–7 weeks old, weighing 150–250 g) and male Institute of Cancer Research mice (6 weeks old, weighing 25–30 g) were obtained from the Charles River Company Korea (Orient, Seoul, Republic of Korea). The animals were allowed to acclimate for one week before the study commenced. Upon arrival, the animals were randomly assigned and housed in groups of two (for rats) or five (for mice) per cage under strictly controlled. The environmental conditions included a 12 h light/12 h dark cycle with a light intensity of 150 to 300 Lux, maintained at 20–25 °C and 48–52% relative humidity. The animals had ad libitum access to food and water.

### 2.3. LC-MS/MS Analysis of MBR in Biological Samples

The concentrations of MBR in the biological samples were determined using a modified UPLC-MS/MS method, as previously described by Chen and Zhang [25] with some modification. Namely, the column length was increased from 5 to 10 cm, the column oven temperature was lowered from 40 to 20 °C, the flow rate was decreased from 0.35 to 0.3 mL/min, the injection volume was increased from 2 to 5 µL, and the initial gradient ratio of the mobile phase was adjusted from 45:55 to 80:20 (*v*/*v*). The analysis was conducted using a Waters XEVO TQ/UPLC system (Waters Corporation, Milford, MA, USA) equipped with a reversed-phase C_18_ column (ACQUITY UPLC BEH, 2.1 mm × 100 mm i.d., particle size: 1.7 µm, Waters, Dublin, Ireland). The mobile phase consisted of 0.1% formic acid in water (A) and acetonitrile (B), and the gradient elution was carried out at a flow rate of 0.3 mL/min. The mass spectrometer was operated in multiple reaction monitoring (MRM) mode with an electrospray ionization interface for positive ions ([M+H]^+^). The various parameters such as capillary voltage, desolvation gas temperature, gas flow rate, and *m*/*z* values for MBR and the IS were optimized. The capillary voltage was set at 1.0 kV, the desolvation gas temperature was maintained at 350 °C, and the gas flow rate was 650 L/h. The *m*/*z* values for MBR and IS were 397.26 → 379.20 (23 and 15 eV for cone voltage and collision energy, respectively) and 373.30 → 305.30 (50 and 30 eV for cone voltage and collision energy, respectively). The analytical data were processed using MassLynx software (version 4.1, Waters Corporation, Milford, MA, USA).

To prepare biological standard samples of MBR in plasma, urine, and the gastrointestinal tract (GI), the MBR stock solution in methanol (20 mg/mL) was serially diluted to make working solutions of MBR in a concentration range of 0.5 to 2000 µg/mL. Each working solution of different concentrations of MBR was added to drug-free plasma, urine, or GI samples to obtain standard samples with final concentration ranges of 0.005 to 200 µg/mL. This enabled the construction of a calibration curve for MBR in each biological sample.

During the sample preparation step for both standard samples of MBR in plasma, urine, and GI, a 50 µL aliquot of the sample was deproteinized by adding 150 µL of methanol for plasma samples or 450 µL for urine and GI samples. Methanol containing 200 ng/mL of the IS was used. After vortexing and centrifugation for 10 min at 12,000 rpm, a 5 µL aliquot of the supernatant was injected into the column for UPLC-MS/MS analysis.

Figure S1 (Supplementary Material) shows typical chromatograms of the stock solution, drug-free biological samples (plasma, urine, and GI samples), standard samples after spiking MBR into drug-free biological samples, and experimental samples obtained after oral administration of MBR at 50 mg (10 mL)/kg to mice. No interfering peaks from endogenous substrates in the drug-free plasma, urine, or GI samples were observed at the elution times of 1.08 and 3.44 min, corresponding to the retention times of MBR and the IS, respectively. Calibration curves were constructed for plasma, urine, and GI samples, displaying reliable responses within the following MBR concentration ranges: 0.005–2.5, 1–200, and 0.1–10 µg/mL, respectively. The lower limits of quantification values of MBR in the plasma, urine, and GI samples were 0.005, 1, and 0.1 µg/mL, respectively. These calibration curves in plasma, urine, and GI samples were constructed for MBR using linear regression analysis, based on the peak area ratios relative to the IS. These values represent the lowest concentrations at which MBR can be accurately quantified in each sample type.

### 2.4. Plasma Protein Binding of MBR Spiked with MBR, MBR-EFA, MBR-NDA, or MBR-PG in Rats and Mice

The plasma protein binding values of MBR in rats and mice were determined using a Rapid Equilibrium Dialysis (RED) device (Thermo Fisher Scientific, Waltham, MA, USA) [26,27]. For the plasma protein binding study using rat plasma, a 100 µL of fresh rat plasma containing MBR, MBR-EFA, MBR-NDA, or MBR-PG (at final concentrations equivalent to 1 µg/mL of MBR) was added to the plasma chamber of the RED device. Afterward, a 300 µL of dialysis buffer solution (i.e., 0.1 M phosphate buffer) was added into the buffer chamber. The samples were then incubated for 4 h at 37 °C, stirring at 250 rpm. After the incubation, a 50 µL of the sample was collected from each chamber (i.e., plasma and buffer chamber, respectively).

Similarly, the plasma protein binding values of MBR, MBR-EFA, MBR-NDA, and MBR-PG in mice were also measured (at final concentrations equivalent to 1 µg/mL of MBR). The other procedures were the same as with the plasma protein binding study using rat plasma. All samples were stored at −80 °C (Revco ULT 1490 D-N-S; Western Mednics, Asheville, NC, USA) for UPLC-MS/MS analysis of MBR.

### 2.5. Pharmacokinetics of MBR after Administration of MBR, MBR-EFA, MBR-NDA, or MBR-PG in Rats

Prior to the experiment, the rats were fasted overnight with free access to water before drug administration. On the day of the experiment, the rats were exposed to diethyl ether anesthesia through inhalation for 5 min. Under anesthesia, cannulation of carotid artery for blood sampling was performed using the previously reported methods [26,27,28].

Upon awakening from anesthesia, the rats were orally administered with MBR, MBR-EFA, MBR-NDA, or MBR-PG at a dose of 30 mg (10 mL)/kg as MBR using gastric gavage tube. Each compound was dissolved in a mixture of ethanol, polyethylene glycol, and distilled water at a ratio of 1:1:3 (*v*/*v*). To administer an equivalent dose of MBR within MBR-EFA, MBR-NDA, and MBR-PG, the molar concentrations of MBR (396.51 g/mol) and each co-former such as EFA (190.2 g/mol) in MBR-EFA, NDA (288.30 g/mol) in MBR-NDA, and PG (129.04 g/mol) in MBR-PG were adjusted. MBR-EFA (491.61 g/mol) consists of a 2:1 ratio of MBR and EFA, MBR-NDA (540.66 g/mol) consists of a 2:1 ratio of MBR and NDA, and MBR-PG (525.55 g/mol) consists of a 1:1 ratio of MBR and PG, respectively. At 0, 5, 15, 30, 60, 120, 180, 240, 360, 480, 600, 720, 960, 1200, and 1440 min after oral administration, approximately 0.12 mL of blood sample was collected from the carotid artery. Each blood sample was immediately centrifuged for 10 min at 12,000 rpm, and a 50 μL of the supernatant (i.e., plasma) was collected. At 24 h after oral administration of each compound, 10 mL of distilled water was used to flush each metabolic cage. Urine samples collected for 24 h were combined with the flushed distilled water. After stirring, a 50 μL of the urine sample was collected. At the same time (i.e., at 24 h), the remaining blood in the body was collected at the same time, and rats were sacrificed by cervical dislocation. The GI including its contents and feces was removed and transferred to a beaker. The GI was then cut into small pieces, after which 100 mL of methanol was added to the beaker. The mixture was manually shaken, and a 50 μL of the supernatant was collected from each beaker. All biological samples were stored at −80 °C for UPLC-MS/MS analysis of MBR.

### 2.6. Pharmacokinetics of MBR after Administration of MBR, MBR-EFA, MBR-NDA, or MBR-PG in Mice

The pharmacokinetic studies using mice were conducted following the previously reported methods [29]. For the intravenous study, MBR was dissolved in a mixture of ethanol, polyethylene glycol, and distilled water at a ratio of 1:1:3 (*v*/*v*). A dose of 5 mg (5 mL)/kg of MBR was intravenously administered to the mice through the tail vein. Approximately 0.12 mL of blood samples were then collected via heart puncture at 0, 5, 15, 30, 60, 120, 180, 240, 360, 480, 600, 720, and 1440 min after MBR administration. A 31-gauge needle was used for the heart puncture to minimize damage to cardiac and pericardial tissues along the needle track, as well as to ensure that the mice were kept alive for several blood collections. The blood samples were immediately centrifuged for 10 min at 12,000 rpm, and a 50 μL of the plasma was collected. At the same time (i.e., at 24 h), the remaining blood in the body was collected, and mice were sacrificed by cervical dislocation. The GI including its contents and feces was removed and transferred to a beaker. The GI was cut into small pieces, and 20 mL of methanol was added to the beaker. The mixture was manually shaken, and a 50 μL aliquot of the supernatant was collected from each beaker.

For the oral study, the mice were fasted overnight with free access to water before drug administration. MBR, MBR-EFA, MBR-NDA, or MBR-PG was dissolved in a mixture of ethanol, polyethylene glycol, and distilled water at a ratio of 1:1:3 (*v*/*v*). The mice were orally administered with MBR, MBR-EFA, MBR-NDA, or MBR-PG at a dose of 50 mg (10 mL)/kg as MBR using a gastric gavage tube. To administer the equivalent doses of MBR within MBR-EFA, MBR-NDA, and MBR-PG, the doses of MBR-EFA, MBR-NDA, and MBR-PG were adjusted by considering the molar concentration of MBR and EFA for MBR-EFA, MBR and NDA for MBR-NDA, and MBR and PG for MBR-PG, as described in the pharmacokinetic study in rats. Approximately 0.12 mL of blood samples were collected via heart puncture at 0, 5, 15, 30, 60, 120, 180, 240, 360, 480, 600, 720, 960, 1200, and 1440 min after oral administration of each compound. The remaining procedures for the oral study were the same as those of the intravenous study. All biological samples were stored at −80 °C for UPLC-MS/MS analysis of MBR.

### 2.7. Tissue Distribution of MBR after Administration MBR, MBR-EFA, MBR-NDA, or MBR-PG in Mice

For the intravenous study, a dose of 5 mg (5 mL)/kg MBR (the same as in the pharmacokinetic study) was intravenously administered to the mice via tail vein. The plasma and tissue samples were collected as described in previous studies [29,30]. At 0.5, 2, 4, 8, and 10 h after MBR administration, as much blood was collected via heart puncture, after which the portal vein was perfused with 0.9% NaCl solution. After perfusion, the heart, lung, liver, stomach, small intestine, large intestine, kidneys, brain, and fat were excised, weighed, and washed with 0.9% NaCl solution. Each tissue was homogenized in a 3-fold volume of 0.9% NaCl solution. After centrifugation at 12,000 rpm for 10 min, a 50 μL aliquot of the supernatant was collected and stored at −80 °C until further analysis using UPLC-MS/MS for MBR.

For the oral study, mice were fasted overnight with free access to water before drug administration. On the day of the experiment, MBR, MBR-EFA, MBR-NDA, or MBR-PG at a dose of 50 mg (10 mL)/kg as MBR (the same as in the pharmacokinetic study) was orally administered to the mice using a gastric gavage tube. To administer the equivalent doses of MBR within MBR-EFA, MBR-NDA, and MBR-PG, the doses of MBR-EFA, MBR-NDA, and MBR-PG were adjusted by considering the molar concentration of MBR and EFA for MBR-EFA, MBR and NDA for MBR-NDA, and MBR and PG for MBR-PG as described in the pharmacokinetic study in rats. The remaining procedures for the collection of blood and tissue samples were the same as in the intravenous study, as described above.

### 2.8. Pharmacokinetic Parameters

Pharmacokinetic parameters, including the total area under the plasma concentration–time curve from time 0 to 24 h (AUC_0–24h_), the total area under the plasma concentration–time curve from time 0 to infinity (AUC_0-inf_), the terminal half-life (terminal t_1/2_), the apparent volume of distribution at a steady state (V_ss_), the total body clearance (CL), and the apparent total body clearance after oral administration (CL/*F*_inf_) were calculated using non-compartmental analysis (PK solver, version 2.1; Scientific). The first peak plasma concentration (*C*_max,1_) and time to reach *C*_max,1_ (*T*_max,1_), as well as the second peak plasma concentration (*C*_max,2_) and time to reach *C*_max,2_ (*T*_max,2_), were directly obtained from the plasma concentration–time data. The absolute *F*_24h_ of MBR was determined by dividing the dose-normalized oral AUC_0–24h_ of MBR or each co-amorphous dispersion of MBR (i.e., MBR-EFA, MBR-NDA, or MBR-PG) by the intravenous AUC_0–24h_ of MBR. The relative *F*_24h_ (or *F*_inf_) was calculated by dividing oral AUC_0–24h_ (or AUC_0-inf_) of each co-amorphous dispersion of MBR by the oral AUC_0–24h_ (or AUC_0-inf_) of MBR.

Based on linear pharmacokinetics, the mean ‘true’ fraction of dose unabsorbed (*F*_unabs_) in the present study can be estimated using the following equation [31]:

GI_24h, oral_ (%) = *F*_unabs_ + [GI_24h, intravenous_ (%) × absolute *F*_24h_ (%)]

### 2.9. Statistical Analysis

A *p* value < 0.05 was considered to be statistically significant using a Tukey’s multiple range test of the Statistical Package of Social Sciences posteriori analysis of variance among the four means for the unpaired data. All data are presented as mean ± standard deviation (S.D.) except for the median (ranges) for *T*_max,1_ and *T*_max,2_.

## 3. Results

### 3.1. Plasma Protein Binding of MBR in Rat or Mouse Plasma Spiked with MBR, MBR-EFA, MBR-NDA, and MBR-PG

The rat plasma protein binding values of MBR were 78.2 ± 7.57% for MBR, 68.2 ± 3.29% for MBR-EFA, 68.7 ± 3.81% for MBR-NDA, and 63.6 ± 10.5% for MBR-PG (equivalent to 1 µg/mL MBR), respectively. There was no significant difference among the four groups of rats. Similarly, mouse plasma protein binding values of MBR were 51.6 ± 18.8%, 60.8 ± 4.05%, 62.3 ± 0.655%, and 59.0 ± 5.54% for MBR, MBR-EFA, MBR-NDA, and MBR-PG (equivalent to 1 µg/mL MBR), respectively. There was also no significant difference among the four groups of mice.

### 3.2. Pharmacokinetics of MBR after Administration of MBR, MBR-EFA, MBR-NDA, or MBR-PG in Rats

**Figure 2:**
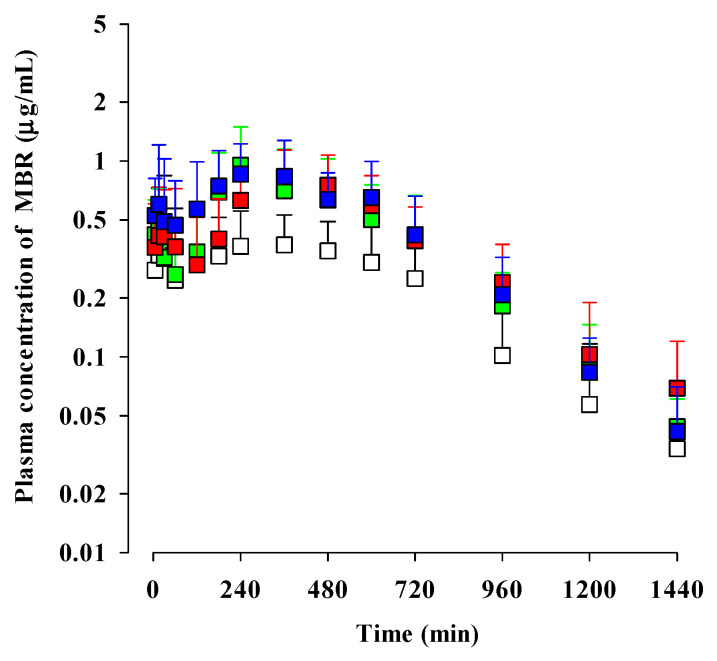
Mean (±S.D.) plasma concentrations of MBR after oral administration of MBR (□; *n* = 14), MBR-EFA (■; *n* = 10), MBR-NDA (■; *n* = 11), and MBR-PG (■; *n* = 10) at a dose of 30 mg/kg as MBR to rats. The “*n*” represents the number of rats used in each group.

The mean plasma concentration–time profiles of MBR after the oral administration of MBR, MBR-EFA, MBR-NDA, and MBR-PG at a dose of 30 mg (10 mL)/kg as MBR to rats are depicted in Figure 2. The corresponding pharmacokinetic parameters of MBR are presented in Table 1. The plasma concentration–time profiles of MBR in all groups of rats showed as distinct peaks at different time points, namely *T*_max,1_ and *T*_max,2_. MBR was detected in plasma at early blood sampling time points (5 or 15 min), and the *T*_max,1_ values were 5 or 15 min in all groups of rats. These findings indicated the rapid absorption of MBR from the GI in all groups. The second peak patterns of MBR were also observed in all groups of rats. There was no significant difference between the *C*_max,2_ at *T*_max,2_ values, as well as *C*_max,1_ at *T*_max,1_ among the four groups of rats.

In terms of elimination, the Ae_0–24h_ values of MBR in MBR-EFA, MBR-NDA, and MBR-PG rats were significantly smaller (34.6, 43.6, and 48.0% decrease, respectively) than that in MBR rats. The GI_24h_ values of MBR, as sum fractions of unabsorbed and biliary excreted MBR, in MBR-EFA, MBR-NDA, and MBR-PG rats were significantly smaller (57.7, 58.0, and 58.2% decrease, respectively) than that in MBR rats. These significant reductions in Ae_0–24h_ and GI_24h_ values of MBR in MBR-EFA, MBR-NDA, and MBR-PG rats were attributed to the slower CL/*F*_inf_ values (20.7, 28.3, and 42.1% decrease, respectively) compared with those in MBR rats.

As a result of the absorption and disposition of MBR, the AUC_0–24h_ values of MBR in MBR-EFA, MBR-NDA, and MBR-PG rats were significantly greater (74.6, 81.2, and 95.4% increase, respectively) than those in MBR rats. The AUC_0-inf_ values of MBR in MBR-EFA, MBR-NDA, and MBR-PG rats were also significantly greater (64.2, 74.1, and 82.8% increase, respectively) than those in MBR rats. Moreover, the relative *F*_24h_ and *F*_inf_ values of MBR were considerably increased in MBR-EFA, MBR-NDA, and MBR-PG rats.

### 3.3. Pharmacokinetics of MBR after Administration of MBR, MBR-EFA, MBR-NDA, or MBR-PG in Mice

**Figure 3:**
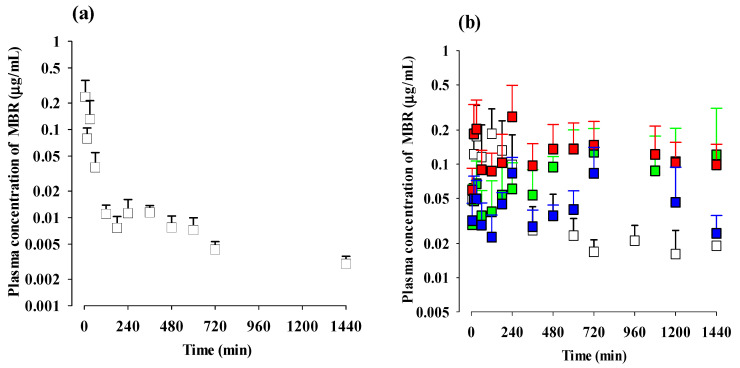
(**a**) Mean (±S.D.) plasma concentrations of MBR after intravenous administration of MBR (□; *n* = 13) at a dose of 5 mg/kg as MBR to mice. (**b**) Mean (± S.D.) plasma concentrations of MBR after oral administration of MBR (□; *n* = 16), MBR-EFA (■; *n* = 15), MBR-NDA (■; *n* = 13), and MBR-PG (■; *n* = 15) at a dose of 50 mg/kg as MBR to mice. The “*n*” represents the number of mice used in each group.

The mean arterial plasma concentration–time profiles and relevant pharmacokinetic parameters of MBR after the intravenous and oral administration of MBR are presented in Figure 3 and Table 2, respectively. The doses were 5 mg (5 mL)/kg and 50 mg (10 mL)/kg of MBR for the intravenous and oral administration, respectively. Following the intravenous administration of MBR, the rapid and extensive distribution of MBR throughout the body were observed, with a volume of distribution of 115 L/kg. The terminal t_1/2_ of MBR was calculated to be 729 min. The biliary excretion of MBR was considered negligible, as indicated by the GI_24h_ value of 2.32% of the intravenously administered dose. After the oral administration of MBR, MBR was detected in plasma as early as the first or second blood sampling time (5 or 15 min) with a *T*_max,1_ of 30 min, indicating its rapid absorption. Following absorption, a second peak of MBR was observed (i.e., *C*_max,2_ of 0.0350 µg/mL at *T*_max,2_ of 420 min). Due to the fluctuations of plasma concentrations in the terminal phase, the terminal t_1/2_, AUC_0-inf_, and *F*_inf_ could not be calculated. Thus, the absolute *F*_24h_ of MBR was calculated to be 30.3%.

The mean arterial plasma concentration–time profiles and relevant pharmacokinetic parameters of MBR after the oral administration of MBR-EFA, MBR-NDA, or MBR-PG are also shown in Figure 3 and Table 2, respectively. Compared with MBR, some pharmacokinetic profiles of MBR changed after the oral administration of MBR-EFA, MBR-NDA, or MBR-PG. Regarding absorption, MBR was detected in the plasma at early blood sampling time points (5 or 15 min), and *T*_max,1_ values were 30 min for all groups of mice. These findings indicated the relatively rapid absorption of MBR from the GI across all groups of mice. The second peak patterns of MBR were observed in all groups of mice, and there was also no significant difference among the groups. However, the GI_24h_ values were significantly smaller (93.6 and 66.4% decrease, respectively) in MBR-EFA and MBR-NDA mice than that in MBR mice. As a result, the AUC_0–24h_ values of MBR in MBR-EFA and MBR-NDA mice were greater (112 and 196% increase, respectively) than those in MBR mice. The absolute *F*_24h_ of MBR, 30.3% in MBR mice, shifted to 54.1 and 70.9% in MBR-EFA and MBR-NDA mice, respectively. Moreover, the relative *F*_24h_ values of MBR in MBR-EFA and MBR-NDA mice were dramatically higher, reaching up to 178 and 234%, respectively. Unlike the MBR in MBR-PG mice, the GI_24h_ value of MBR was not significantly different from that in MBR mice, which likely explains a little change in AUC_0–24h_ (15.1% increase) and absolute *F*_24h_ (15.2% increase), as well as a relative *F*_24h_ of 115% compared with those in MBR mice. Due to the fluctuations in plasma concentrations during the terminal phase, the terminal t_1/2_, AUC_0-inf_, and *F*_inf_ could not be calculated.

### 3.4. Tissue Distribution of MBR after Administration of MBR, MBR-EFA, MBR-NDA, or MBR-PG in Mice

**Figure 4:**
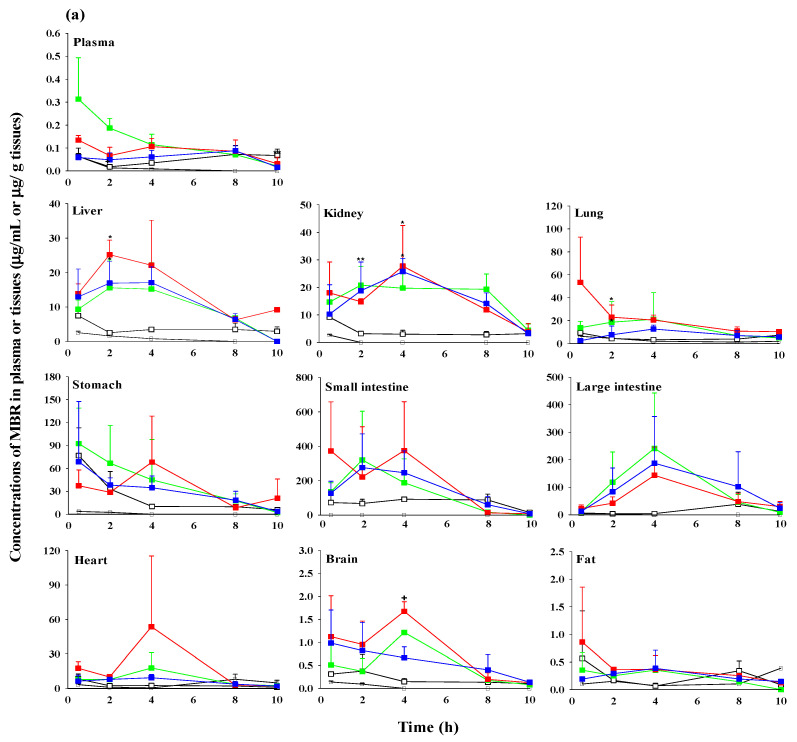

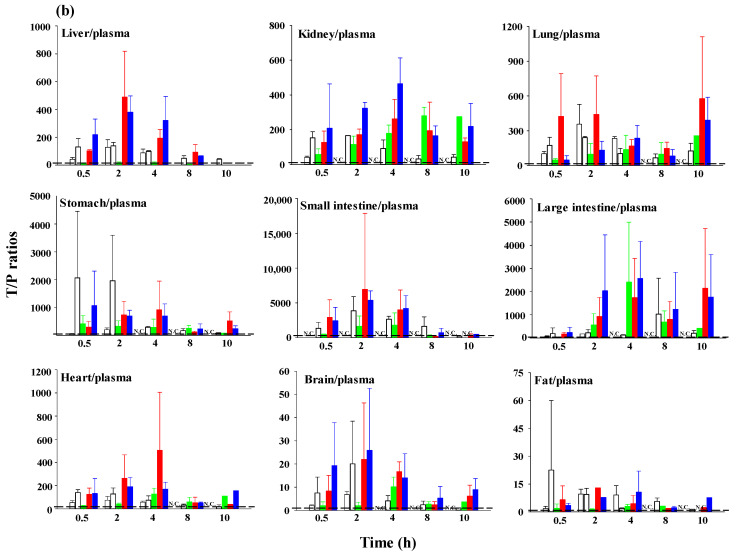
Mean (±S.D.) (**a**) concentrations (µg/mL in plasma and µg/g tissue) and (**b**) tissue/plasma ratios of MBR (▩; *n* = 15 for intravenous and □; *n* = 15 for oral administration), MBR-EFA (■; *n* = 15), MBR-NDA (■; *n* = 15), and MBR-PG (■; *n* = 15) in various tissues at 0.5, 2, 4, 8, and 10 h after intravenous administration of MBR (5 mg/kg as MBR) and oral administration (50 mg/kg as MBR) of MBR, MBR-EFA, MBR-NDA, and MBR-PG to mice. * MBR-NDA and MBR-PG mice were significantly different (*p* < 0.05) from MBR mice. ** MBR-EFA and MBR-PG mice were significantly different (*p* < 0.05) from MBR mice. + MBR-NDA mice were significantly different (*p* < 0.05) from MBR mice. The “*n*” represents the number of mice used in each group.

The concentrations and tissue-to-plasma ratios (T/P ratios) of MBR in plasma and/or various tissues at 0.5, 2, 4, 8, and 10 h after administration of MBR, MBR-EFA, MBR-NDA, and MBR-PG are presented in Figure 4 and Table S1 (Supplementary Material). The AUC_0–10h_ of MBR in various tissues were also calculated as shown in Table 3.

After the intravenous administration of MBR, MBR showed extensive distribution to the liver, lung, stomach, and heart, because the T/P ratios of MBR were above 1 in most of these tissues (Figure 4 and Table S1 (Supplementary Material)). In contrast, after the oral administration of MBR, MBR exhibited very extensive distribution in the GI including the stomach, small intestine, and large intestine compared with other tissues in all groups of mice. Although the T/P ratios of MBR varied depending on the tissue extraction time (i.e., sampling time), most tissues exhibited T/P ratios considerably above 1, indicating the significant and favorable tissue distribution of MBR. Even in the brain or fat, where MBR distribution was relatively lower than in other tissues, the T/P ratios were still higher than 1. Interestingly, the distribution rate of MBR to the brain and fat appeared relatively slower, with lower concentrations and smaller T/P ratios of approximately 1 compared with other tissues. This suggest that the distribution rate of MBR to different tissues varied over time, particularly at 0.5, 2, 4, 8, and 10 h after its oral administration. During absorption, MBR was initially exposed in the stomach and small intestines, after which it rapidly distributed to highly perfused organs such as the liver, heart, lung, and kidneys. Concentrations of MBR in these organs were higher at 0.5 h compared with 10 h, indicating the rapid distribution and elimination of MBR in these tissues.

Different distribution patterns of MBR were observed among the four groups of mice. Compared with MBR mice, the tissue distribution of MBR (i.e., MBR concentrations and T/P ratios) appeared to be higher in most tissues of MBR-EFA, MBR-NDA, or MBR-PG mice. Particularly, in MBR-NDA mice, MBR concentrations in the liver, kidneys, and brain at 2, 4, and 8 h were significantly greater (896, 325, and 1036% increase, respectively) than in MBR mice, although the S.D. values were relatively large. This result suggests that MBR-NDA might enhance MBR exposure in several tissues such as the liver, kidneys, and brain.

To further assess MBR exposure in each tissue, the AUC_0–10h_ (µg min/g tissue) of MBR was calculated. In the intravenous study, the AUC_0–10h_ (µg min/g tissue) of MBR was calculated only in the liver, lung, and heart, as MBR concentrations were not detected beyond 4 or 6 h in other tissues. In the oral study, the AUC_0–10h_ of MBR in the kidneys of MBR mice was significantly smaller than those in other three groups of mice. In MBR-NDA mice, the AUC_0–10h_ values of MBR in the liver, lung, and kidneys were significantly greater (289, 289, and 313% increase, respectively) than those in MBR mice.

## 4. Discussion

The *F* value, which represents drug exposure, is a critical parameter in determining pharmacokinetic feasibility and drug efficacy [32,33,34,35,36,37,38,39], as it influences the time-course of efficacy and toxicity. In the assessment of orally administered compounds, the in vivo *F* value and absorption rate are of considerable importance, along with in vitro solubility and dissolution rate [40]. Traditionally, rats have been used as representative models in toxicology and pharmacology, especially in pharmacokinetics research, due to their convenience and similarity to human physiology. However, mice are becoming increasingly popular as an attractive model. This shift is attributed to certain limitations of rat models, including challenges in obtaining sufficient compounds, a narrower range of disease models compared with mice, and difficulties in extrapolating preclinical data to humans through animal scale-up systems [41,42].

A recommended commercial dose of 50 mg MBR (Myrbetriq^®^) for once daily administration is approved [4]. It was also reported that mirabegron was generally well tolerated at 20–300 mg once daily [43] and 50–400 mg once daily [44]. According to the human equivalent dose (HED) equation, the extrapolated doses of MBR were 2.23–26.8 mg/kg in rats and 4.46–53.6 mg/kg in mice from a 20–300 mg human dose and 4.46–35.7 mg/kg in rats and 8.93–71.4 mg/kg in mice from a 50–400 mg human dose, assuming 70 kg of human body weight [4,45].

In pre-clinical levels, the slight side effect (i.e., decreased movement) of MBR was observed in both rats and mice when 250 mg/kg and 100 mg/kg of MBR was administered in rats and mice, respectively [46]. These doses of MBR were 8.33-fold and 2-fold higher than the used doses, at 30 mg/kg and 50 mg/kg in rats and mice in this study, respectively. Since the used doses in this study (30 mg/kg in rats and 50 mg/kg in mice) were lower than the doses causing even slight side effects (250 mg/kg in rats and 100 mg/kg in mice [46]), the used doses in this study are appropriate within safe dose ranges. In addition, a 2.5 mg/mL of MBR showed <82% of cytotoxicity in in vitro HeLa cells [47]. The plasma concentrations of MBR, especially *C*_max_ values of 0.491–1.09 µg/mL in rats and 0.0672–0.261 µg/mL in mice, were lower than 2.5 mg/mL showing in vitro cytotoxicity. This result indicated that all plasma concentrations of MBR might be included under the cytotoxic concentration. The doses used in this study were chosen considering the previously reported information.

MBR has a pKa of 8.00 and a partition coefficient (log P) of 2.89 [7,48]. Additionally, MBR exhibits a low *F* of approximately 29% [5,6]. This limited *F* can be primarily attributed to its poor solubility (0.082 mg/mL; [6]) and low permeability (1.68−1.83 × 10^−6^ cm/s in Caco-2 cells; [9]), which hinder its dissolution and absorption in the GI. These properties resulted in incomplete and variable absorption, leading to the low *F* values ranging from 18 to 34% of the oral dose [49,50]. After the absorption phase, various factors, such as distribution, metabolism, and excretion, can contribute to the low *F* of MBR. MBR is extensively metabolized via cytochrome P450 (CYP) enzymes, including CYP2D6 and CYP3A4, as well as uridine 5′-diphospho-glucuronosyltransferases (UGTs), such as UGT2B7, which further contributes to the first-pass effect and low *F* of MBR [7,46,51]. MBR and its metabolites formed via hepatic or intestinal metabolism are eliminated through kidneys and bile [46,52]. Previous rat studies indicated that approximately 10–20% of intravenously administered MBR undergoes biliary and urinary excretion [46,53]. Approximately 37% of the unchanged MBR plus its metabolites are excreted in the urine, while the remainder is excreted in the bile [46,52]. Specific glucuronidation forms of MBR, such as M11, M12, M15, and M17, account for 3.2, 1.4, 0.6, and 2.0% of the Ae_0–24h_ values, respectively [8], while unchanged MBR is also excreted through urinary excretion. Additionally, MBR demonstrates a high protein binding capacity of approximately 71.0% [7,46,52], which can limit its distribution and availability in the systemic circulation. However, despite this limitation, MBR exhibits extensive distribution throughout the body [10,50]. This highlights the importance of improving the low solubility and permeability of MBR, which might increase *F*. Although the crystalline solid form of MBR is marketed and available [53], the further development of MBR formulations is needed to enhance *F* in its formulations as the ongoing research and development effects.

In a recent report by An et al. [6] and a corresponding patent [24], several co-amorphous formulations of MBR, such as MBR-EFA, MBR-NDA, or MBR-PG, were developed to address the low solubility of MBR and the restrictions associated with PEG800 use during the formation process. The solubility and pKa of each co-former were 147, 90.52, and 100 g/L and −1.46, 1.35, and 3.32 in EFA, NDA, and PG, respectively. Considering the pKa of MBR, each pKa difference between co-former and MBR was 9.46, 6.70, and 4.68 in EFA, NDA, and PG, respectively. Thus, the solubility and pKa difference supported that the three co-factors are appropriately chosen to form co-amorphous dispersions of MBR. The thermodynamic stability is another critical factor in enhancing the solubility in co-amorphous dispersions. In particular, the stronger acidity of co-formers can lead the higher *T_g_* of the co-amorphous dispersions [6]. In the co-amorphous dispersions used in this study, the thermodynamic stability of each co-amorphous dispersion increased according to *T_g_* values as follows: MBR amorphous (*T_g_* = 37.74 °C) < MBR-PG (*T_g_* = 93.34 °C) < MBR-NDA (*T_g_* = 201.34 °C) < MBR-EFA (*T_g_* = 255.77 °C). This result indicated that the increase in thermodynamic stability and the *T_g_* of co-amorphous dispersions matched well. These results are consistent in that the stronger acidity of a co-former shows a higher *T_g_* of the co-amorphous dispersion. Taking the above points into consideration, the water-solubility and pKa of the co-former are critical factors in making co-amorphous dispersions of MBR. In our results, the solubility of MBR-EFA, MBR-NDA, and MBR-PG in water and pH 6.8 were greatly improved compared with the crystalline solid state of MBR, and deliquescence of MBR-EFA, MBR-NDA, and MBR-PG did not occur at a relative humidity of 33 to 93% [24]. The primary objective of this study is to assess the pharmacokinetic feasibility of these co-amorphous formulations at the preclinical level, employing both rats and mice simultaneously for the first time.

After the oral administration of MBR-EFA, MBR-NDA, and MBR-PG to rats, the significantly increased AUC values of MBR indicated that MBR exposures were enhanced compared with MBR rats. The relative *F*_24h_ values of MBR, which were 146, 143, and 195% in MBR-EFA, MBR-NDA, and MBR-PG rats, paralleled the increases in systemic exposure compared with the MBR rats (Table 1). While the absorption rate of MBR remained unchanged, as made evident by comparable *C*_max,1_ at *T*_max,1_ among all groups of rats, the extents of the absorption and systemic exposure of MBR were notably increased in MBR-EFA, MBR-NDA, and MBR-PG rats compared with MBR rats. These results suggest that the systemic exposure of MBR could be accelerated due to the enhanced absorption of MBR in MBR-EFA, MBR-NDA, and MBR-PG rats compared with the MBR rats. It is important to note that GI_24h_, which represents the sum fraction of the unabsorbed dose and biliary excreted dose 24 h after oral administration, does not provide an exact calculation of the unabsorbed percentage of the oral dose [31]. Therefore, unabsorbed fraction (*F*_unabs_) after the oral administration of MBR to rats was calculated based on a reported equation [31]. The calculated ‘*F*_unabs_’ was 18.6, 5.85, 5.86, and 4.80% for MBR, MBR-EFA, MBR-NDA, and MBR-PG, respectively. In the process of calculating ‘*F*_unabs_’, the values of the pharmacokinetic parameters (i.e., GI_24h, oral_, GI_24h, intravenous_, and absolute *F*_24h_) were used.

Here, the GI_24h, oral_ values for MBR, MBR-EFA, MBR-NDA, and MBR-PG rats were determined to be 20.6, 8.71, 8.66, and 8.62%, respectively (Table 1). Since the GI_24h, intravenous_ was not directly obtained in this study, an assumed value of 6.75% was used for MBR rats based on a previous report [46]. This value was derived from the biliary excretion of unchanged MBR, which was found to be a quarter of the biliary excretion of ^14^C-radiolabelled MBR, which in turn corresponded to 27% of intravenous ^14^C-radiolabelled MBR. The estimated absolute *F*_24h_ values for MBR, MBR-EFA, MBR-NDA, and MBR-PG rats were calculated to be 29.0, 42.4, 41.5, and 56.6%, respectively, based on the absolute *F*_24h_ of MBR and the relative *F*_24h_ of MBR-EFA, MBR-NDA, and MBR-PG. The extent of oral absorption of MBR was found to be enhanced in the MBR-EFA, MBR-NDA, and MBR-PG rats compared with the MBR rats: 81.4, 94.2, 94.1, and 95.2% of the dose might be absorbed in rats. In other words, co-amorphous MBR-EFA, MBR-NDA, and MBR-PG enhanced the absorption of MBR compared with MBR itself.

Following absorption, double-peak phenomena were observed in the plasma concentration–time profiles of MBR in rats (Figure 2 and Table 1). Several mechanisms could explain the appearance of second peaks in MBR, MBR-EFA, MBR-NDA, and MBR-PG rats [54]. Firstly, the double peaks may be attributed to the enterohepatic circulation of MBR in rats, as previously reported [46]. Comparable *C*_max,2_ and *T*_max,2_ values among four groups of rats suggest that the biliary excretion and reabsorption from intestine to blood might not be changed. This is further supported by the similar terminal t_1/2_ of MBR, ranging from 228 to 279 min, among four groups of rats (Table 1). Secondly, the appearance of double peaks can be explained by solubility-limited absorption, considering the low solubility of MBR. Similar second peak profiles at approximately 4–8 h have been reported for levodopa and doxycycline [55,56]. Thirdly, the reversible distribution between tissue and plasma can play a role in the occurrence of second peaks. In other words, MBR may undergo reversible movement between tissues and plasma over time. Despite its high plasma protein binding value (i.e., 63.6–78.2% across all groups of rats in this study and 71.0%, as the previously reported value [53]), MBR is known to rapidly and extensively distribute to extravascular sites or tissues. For instance, multiple peaks have been observed for trimethoprim and sulfadiazine due to their extensive distribution and reversible movement between tissues and systemic circulation [56,57].

In the intravenous study using mice, it was observed that the V_ss_ of MBR (115 L/kg in Table 2) was higher than the reported plasma volume values of 1 mL in 20 g mice [58]. This indicates that MBR was extensively distributed throughout the body. Similar to the rats, the plasma protein binding of MBR in mice was consistently high (ranging from 51.6 to 62.3%) across all groups of mice. Regarding elimination, a previous study reported that the renal excretion of MBR in its unchanged form was considerable [46]. After the oral administration of ^14^C-radiolabelled MBR at a dose of 160 mg as MBR, 55% of the dose was excreted in the urine, in which 25% was in the unchanged form of MBR [8,53]: 13.8% of the ^14^C-radiolabelled MBR as a parent form of MBR and 31.2% as metabolites were excreted into the urine [8,53]. Additionally, the contribution of the gastrointestinal excretion (including biliary excretion) of unchanged MBR to its CL appeared to be negligible, as evidenced by the low GI_24h_ value of only 2.32% of the intravenous dose (Table 2). Hence, CL_R_ represents the main elimination pathway for MBR and its metabolites, as reported previously [8,46]. For example, the Ae_0–24h_ values of the glucuronidated forms of MBR, specifically M11, M12, M15, and M17, were found to be 3.2, 1.4, 0.6, and 2.0%, respectively [8], suggesting that the urinary excretion of MBR occurs in the forms of MBR itself and its glucuronidated metabolites.

In the oral study, the extent of the oral absorption of MBR was found to be enhanced in the MBR-EFA, MBR-NDA, and MBR-PG mice compared with the MBR mice. The process from the oral administration of MBR and its co-amorphous dispersions to the absorption was visualized in Figure 1b. To quantitatively compare the extent of absorption, the ‘*F*_unabs_’ was estimated using an equation previously used with rats [31]. In MBR mice, the GI_24h, intravenous_ value was 2.32%, and the absolute *F*_24h_ was 30.3% (Table 2). In MBR-EFA, MBR-NDA, and MBR-PG mice, the GI_24h, oral_ values were 1.24, 6.54, and 10.9%, and the absolute *F*_24h_ values were 54.1, 70.9, and 34.9%, respectively. Based on these values, the estimated ‘*F*_unabs_’ values for MBR, MBR-EFA, MBR-NDA, and MBR-PG mice were determined to be 18.8, −0.0151, 4.90, and 10.1%, respectively. These results clearly indicate that MBR absorption was enhanced in the MBR-EFA and MBR-NDA mice compared with the MBR mice. Therefore, the greater AUC and higher *C*_max,2_ values were observed in MBR-EFA and MBR-NDA mice, representing the increased systemic exposure of MBR in these mice compared with MBR mice. These results suggest that MBR-EFA and MBR-NDA could be considered as promising co-amorphous formulations of MBR to enhance its oral *F*.

Pharmacokinetics involves the study of the plasma concentration–time profiles of drugs in the bloodstream and tissues, encompassing factors such as absorption rate, drug concentration, and the duration of drug presence at specific locations within the body. Upon entering the systemic circulation or reaching target tissues, a compound such as a drug interacts with various proteins (e.g., receptors, enzymes, or transporters) to initiate biological events leading to a pharmacological response [34,37,39]. The distribution of a given compound in different tissues can explain its tissue-specific pharmacological response, and the relationship between the plasma and tissue levels of a drug is commonly used to optimize dosage regimens and understand pharmacodynamic outcomes [33,36,37,59]. The tissue distribution characteristics of a compound also play a crucial role in its delivery to specific target organs and their affinity for those organs [42,60]. In the case of MBR, after intravenous administration, its distribution to various tissues occurred at different rates and affinities, which influenced its pharmacological activity in the alimentary canal and excretory organs [61]. This distribution pattern of MBR in different tissues is associated with its pharmacological activity. When the concentration of MBR at its target site is sufficiently high, the formulations of MBR exhibit in vivo efficacy. Thus, understanding the pharmacokinetic properties, including tissue distribution, can provide valuable information for selecting appropriate in vivo disease models and further evaluating in vitro potency.

After the oral administration of MBR, the MBR was initially distributed to the stomach during the absorption process and subsequently underwent extensive distribution to highly perfused organs, such as the small intestine, large intestine, heart, lung, and kidneys. According to the AUC_0–10h_ values in tissues (Table 3), MBR exhibited higher distribution in the stomach, small intestine, and large intestine compared with other tissues (e.g., lung, liver, kidneys, heart, brain, and fat) in the MBR mice. Although the distribution pattern varied depending on the tissue extraction time, T/P ratios above 1 were observed in the stomach, small intestine, large intestine, heart, lung, liver, kidneys, brain, and fat. The distribution of MBR in the brain and fat tissues was relatively slower, as indicated by lower concentrations, with smaller T/P ratios around 1, compared with other tissues, and a longer time to peak MBR concentrations. Interestingly, the T/P ratios for MBR increased from 0.5 to 10 h, indicating that the elimination rate of MBR in plasma was faster compared with other tissues. Similar tissue distribution patterns were observed in MBR-EFA, MBR-NDA, and MBR-PG mice. When comparing the oral administration of MBR between MBR and other co-amorphous formulations, high tissue concentrations and T/P ratios were observed in the heart, lung, liver, stomach, small intestine, large intestine, kidneys, brain, and fat in co-amorphous formulation groups of mice. These results suggest that MBR and various formulations exhibited good distribution to most tissues with high affinities.

## 5. Conclusions

The increased water solubility of MBR in MBR-EFA, MBR-NDA, and MBR-PG enhanced the systemic exposures of MBR (i.e., 115–234% of relative *F* values) in co-amorphous dispersions of MBR. Additionally, the tissue exposure of MBR to the liver, kidneys, and lungs were enhanced in those co-amorphous dispersions of MBR compared with MBR itself. Although a slight difference between rats and mice was observed, our findings suggest that the potential of MBR-EFA, MBR-NDA, and MBR-PG leads to enhanced MBR exposure in both plasma and tissues, thereby possibly improving therapeutic efficacy. As a further investigation, the clinical studies with the appropriate dosage regimens will be required to elucidate the improved therapeutic efficacy of the developed formulations. Our results in non-clinical studies can provide basic information to predict and/or estimate the appropriate dosage regimens for clinical levels.

## Figures and Tables

**Table 1:** Mean (±S.D.) pharmacokinetic parameters of MBR after oral administration of MBR-EFA, MBR-NDA, and MBR-PG at a dose of 30 mg/kg as MBR to rats.

Parameters	MBR Rats (*n* = 14)	MBR-EFA Rats (*n* = 10)	MBR-NDA Rats (*n* = 11)	MBR-PG Rats (*n* = 10)
Body weight (g)	199 ± 37.4	217 ± 50.3	240 ± 40.2	245 ± 44.1
Terminal t_1/2_ (min)	279 ± 143	240 ± 94.7	252 ± 98.1	228 ± 75.7
*C*_max,1_ (μg/mL)	0.491 ± 0.498	0.518 ± 0.224	0.577 ± 0.385	0.711 ± 0.570
*T*_max,1_ (min) ^a^	15 (5–180)	15 (5–180)	15 (5–60)	5 (5–15)
*C*_max,2_ (μg/mL)	0.491 ± 0.175	1.00 ± 0.512	0.977 ± 0.255	1.09 ± 0.416
*T*_max,2_ (min) ^a^	420 (180–720)	240 (180–600)	360 (240–480)	360 (180–600)
AUC_0–24h_ (μg min/mL) *	303 ± 84.7	529 ± 225	549 ± 122	592 ± 203
AUC_0-inf_ (μg min/mL) *	332 ± 90.3	545 ± 221	578 ± 123	607 ± 210
CL/*F*_inf_ (mL/min/kg) **	96.2 ± 24.4	76.3 ± 30.3	69.0 ± 18.2	55.7 ± 20.9
GI_24h_ (% of dose) *	20.6 ± 6.88	8.71 ± 1.09	8.66 ± 2.32	8.62 ± 3.22
Ae_0–24h_ (% of dose) *	16.2 ± 6.61	10.6 ± 5.12	9.13 ± 4.02	8.42 ± 4.95
Relative *F*_24h_ (%)		146	143	195
Relative *F*_inf_ (%)		138	137	183

The “*n*” represents the number of rats used in each group. ^a^ The data are presented as median values (ranges). * MBR rats were significantly different (*p* < 0.05) from the MBR-EFA, MBR-NDA, and MBR-PG rats. ** MBR rats were significantly different (*p* < 0.05) from the MBR-NDA and MBR-PG rats.

**Table 2:** Mean (±S.D.) pharmacokinetic parameters of MBR after intravenous administration of MBR at a dose of 5 mg/kg as MBR and oral administration of MBR, MBR-EFA, MBR-NDA, and MBR-PG at a dose of 50 mg/kg as MBR to mice, respectively.

	Intravenous	Oral			
Parameters	MBR Mice (*n* = 13)	MBR Mice (*n* = 16)	MBR-EFA Mice (*n* = 15)	MBR-NDA Mice (*n* = 13)	MBR-PG Mice *(n* = 15)
Body weight (g)	30.3 ± 0.651	31.6 ± 2.09	31.0 ± 1.13	28.7 ± 0.484	28.6 ± 0.625
Terminal t_1/2_ (min)	729				
*C*_max,1_ (μg/mL)		0.176	0.0672	0.203	0.0495
*T*_max,1_ (min) ^a^		30	30	30	30
*C*_max,2_ (μg/mL)		0.0350	0.136	0.261	0.0835
*T*_max,2_ (min) ^a^		420	600	240	240
AUC_0–24h_ (μg min/mL)	20.5	62.2	132	184	71.6
AUC_0-inf_ (μg min/mL)	23.7				
CL (L/min/kg)	0.211				
V_ss_ (L/kg)	115				
GI_24h_ (% of dose) *	2.32 ± 1.31	19.5 ± 13.1	1.24 ± 0.510	6.54 ± 1.11	10.9 ± 7.52
Absolute *F*_24h_ (%) ^b^		30.3	54.1	70.9	34.9
Relative *F*_24h_ (%)			178	234	115

Parameters are calculated using the average plasma concentrations from all mice in each group. ^a^ The data are presented as median values (ranges). ^b^ The analyses were conducted under the assumption that the intravenous and oral administration doses of MBR follow linear pharmacokinetics. * In the oral study, the MBR mice was significantly different (*p* < 0.05) from the MBR-EFA and MBR-NDA mice.

**Table 3:** Mean (±S.D.) AUC_0–10h_ (µg min/g tissue) of MBR in various tissues after intravenous administration of MBR (5 mg/kg as MBR) and oral administration (50 mg/kg as MBR) of MBR, MBR-EFA, MBR-NDA, and MBR-PG to mice.

	Intravenous	Oral			
	MBR Mice (*n* = 15)	MBR Mice (*n* = 15)	MBR-EFA Mice (*n* = 15)	MBR-NDA Mice (*n* = 15)	MBR-PG Mice (*n* = 15)
Liver *	318 ± 91.5	2062 ± 420	4781 ± 2601	8025 ± 2412	5294 ± 1651
Kidney **		2244 ± 585	9732 ± 1180	9272 ± 3514	9326 ± 1831
Small intestine		41,729 ± 7923	67,826 ± 39,305	91,307 ± 64,023	84,272 ± 44,757
Large intestine		8843 ± 7866	53,914 ± 30,115	36,058 ± 17,154	55,281 ± 27,169
Stomach		11,103 ± 1785	23,366 ± 13,827	15,663 ± 6123	13,809 ± 8054
Lung +	1567 ± 18.2	2764 ± 496	6657 ± 3334	10,749 ± 2373	4442 ± 425
Heart	313 ± 57.5	1562 ± 471	3504 ± 2739	9371 ± 7647	2776 ± 1038
Brain		102 ± 31.5	224 ± 92.9	435 ± 51.8	325 ± 73.2
Fat		102 ± 55.6	71.4 ± 61.7	142 ± 104	117 ± 45.5

The “*n*” represents the number of mice used in each group. * MBR-NDA mice were significantly different (*p* < 0.05) from MBR mice. ** MBR-EFA, MBR-NDA, and MBR-PG mice were significantly different (*p* < 0.05) from MBR mice. + MBR-NDA mice were significantly different (*p* < 0.05) from MBR and MBR-PG mice.

## Data Availability

Not applicable.

## Supplementary Materials

The following supporting information can be downloaded at: https://www.mdpi.com/article/10.3390/pharmaceutics15092277/s1, Figure S1: Representative chromatograms of MBR (upper) and IS (lower) (a) stock solution of 0.01 µg/mL MBR and IS; (b) drug-free mouse plasma; (c) mouse plasma standard of 1 µg/mL MBR; (d) mouse plasma sample at 30 min after oral administration of 50  mg/kg MBR; (e) drug-free mouse urine; (f) mouse urine standard of 10 µg/mL MBR; (g) mouse urine sample collected at 24 h after oral administration of 50  mg/kg MBR; (h) drug-free mouse GI; (i) mouse GI standard of 1 µg/mL MBR; (j) mouse GI sample collected at 24 h after oral administration of 50  mg/kg MBR; Table S1: Mean (±S.D.) concentrations and T/P ratios of MBR in plasma and various tissues at 0.5, 2, 4, 8, and 10 h after intravenous administration (5 mg/kg as MBR) and oral administration (50 mg/kg as MBR) of MBR or various formulations of MBR to mice. The units of MBR concentrations in plasma and tissues are presented in µg/mL and µg/g tissue, respectively.
