# Supplementary material for: Improved Pharmacokinetic Feasibilities of Mirabegron-1,2-Ethanedisulfonic Acid, Mirabegron-1,5-Naphthalenedisulfonic Acid, and Mirabegron-L-Pyroglutamic Acid as Co-Amorphous Dispersions in Rats and Mice
Source: Pharmaceutics. 2023 Sep 4;15(9):2277. doi: 10.3390/pharmaceutics15092277 (PMC10536516; doi:10.3390/pharmaceutics15092277)
Supplement: Supplementary file 1 [file pharmaceutics-15-02277-s001.zip › pharmaceutics-2564931-supplementary.pdf]

Supplementary Material

Figure S1. Representative chromatograms of MBR (upper) and IS (lower) (**a**) stock solution of 0.01 μg/mL MBR and IS; (**b**) drug-free mouse plasma; (**c**) mouse plasma standard of 1 μg/mL MBR; (**d**) mouse plasma sample at 30 min after oral administration of 50 mg/kg MBR; (**e**) drug-free mouse urine; (**f**) mouse urine standard of 10 μg/mL MBR; (**g**) mouse urine sample collected at 24 h after oral administration of 50 mg/kg MBR; (**h**) drug-free mouse GI; (**i**) mouse GI standard of 1 μg/mL MBR; (**j**) mouse GI sample collected at 24 h after oral administration of 50 mg/kg MBR.

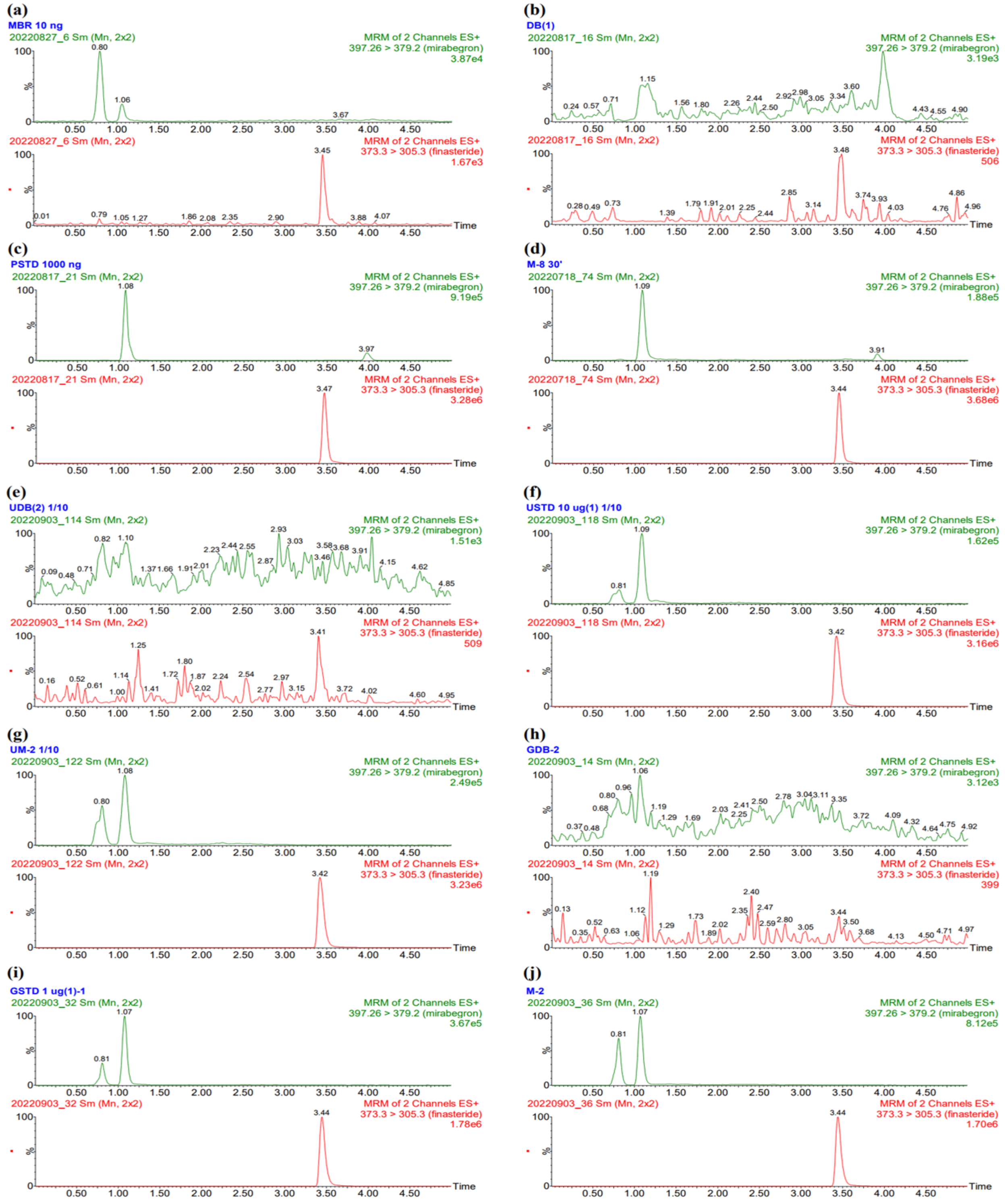

Table S1. Mean (± S.D.) concentrations and T/P ratios of MBR in plasma and various tissues at 0.5, 2, 4, 8, and 10 h after intravenous administration (5 mg/kg as MBR) and oral administration (50 mg/kg as MBR) of MBR or various formulations of MBR to mice. The units of MBR concentrations in plasma and tissues are presented in μg/mL and μg/g tissue, respectively.

	Intravenous	Oral			
**Parameter**	**MBR**	**MBR**	**MBR-EFA**	**MBR-NDA**	**MBR-PG**
**0.5 h**					
Plasma	0.0635 ± 0.0127	0.0633 ± 0.0361	0.313 ± 0.180	0.135 ± 0.0198	0.0589 ± 0.0126
Liver	2.53 ± 0.462	7.47 ± 4.97	9.33 ± 2.66	13.9 ± 2.84	12.9 ± 8.10
	(41.8 ± 14.1)	(131 ± 59.8)	(34.5 ± 14.7)	(102 ± 8.36)	(219 ± 112)
Kidney	2.67 ± 0.231	9.20 ± 4.61	14.7 ± 2.57	18.0 ± 11.2	10.3 ± 10.7
	(42.8 ± 6.16)	(154 ± 34.7)	(58.9 ± 31.4)	(128 ± 65.0)	(209 ± 255)
Small intestine	N.D.	72.5 ± 52.3	135 ± 52.5	372 ± 286	127 ± 69.8
	(N.C.)	(1353 ± 863)	(464 ± 89.0)	(2901 ± 2539)	(2434 ± 1920)
Large intestine	4.00 ± 1.70	6.67 ± 4.41	11.7 ± 8.10	22.8 ± 13.1	12.4 ± 8.65
	(70.4 ± 26.4)	(191 ± 244)	(35.9 ± 4.23)	(166 ± 78.3)	(242 ± 221)
Stomach	3.60 ± 0.400	76.8 ± 36.3	92.4 ± 46.4	37.3 ± 20.6	68.6 ± 78.9
	(58.0 ± 11.2)	(2066 ± 2394)	(412 ± 304)	(296 ± 201)	(1066 ± 1242)
Lung	6.40 ± 0.400	9.33 ± 2.89	13.9 ± 5.46	53.3 ± 39.6	2.53 ± 1.29
	(103 ± 18.4)	(174 ± 70.3)	(47.7 ± 12.7)	(424 ± 368)	(48.0 ± 36.2)
Heart	3.33 ± 0.611	8.40 ± 4.21	7.73 ± 3.78	17.6 ± 5.66	6.40 ± 5.09
	(54.5 ± 16.8)	(141 ± 24.4)	(25.9 ± 4.21)	(125 ± 53.7)	(133 ± 129)
Brain	0.138 ± 0.0195	0.312 ± 0.0516	0.508 ± 0.435	1.12 ± 0.891	0.983 ± 0.721
	(2.13 ± 0.246)	(7.50 ± 6.88)	(2.03 ± 1.64)	(8.31 ± 6.76)	(19.3 ± 18.5)
Fat	0.102 ± 0.0600	0.564 ± 0.862	0.354 ± 0.318	0.859 ± 1.00	0.191 ± 0.0223
	(1.84 ± 1.17)	(22.5 ± 37.5)	(1.86 ± 2.39)	(6.57 ± 7.45)	(3.42 ± 1.19)
**2 h**					
Plasma	0.0135 ± 0.0400	0.0182 ± 0.0024	0.187 ± 0.0421	0.0667 ± 0.0367	0.0490 ± 0.0297
Liver*	1.60 ± 0.400	2.53 ± 0.611	15.6 ± 8.03	25.2 ± 4.21	16.9 ± 6.21
	(128 ± 54.2)	(139 ± 25.1)	(84.4 ± 41.3)	(488 ± 331)	(380 ± 117)
Kidney**	N.D.	N.D.	20.8 ± 6.73	14.8 ± 1.06	18.8 ± 10.4
	(N.C.)	(N.C.)	(115 ± 49.0)	(172 ± 32.4)	(324 ± 34.1)
Small intestine	N.D.	66.9 ± 24.8	321 ± 283	221 ± 292	276 ± 196
	(N.C.)	(3864 ± 2031)	(1635 ± 1479)	(6923 ± 10961)	(5344 ± 1378)
Large intestine	N.D.	3.87 ± 1.89	118 ± 110	42.1 ± 23.1	83.6 ± 86.4
	(N.C.)	(226 ± 144)	(560 ± 489)	(932 ± 815)	(2029 ± 2417)
Stomach	2.53 ± 0.231	32.9 ± 23.1	66.7 ± 49.6	28.6 ± 3.11	38.2 ± 9.33
	(200 ± 68.3)	(1959 ± 1639)	(327 ± 195)	(730 ± 496)	(691 ± 212)
Lung	4.67 ± 1.97	4.40 ± 0.693	18.7 ± 17.9	23.1 ± 10.6	7.73 ± 7.55
	(356 ± 171)	(242 ± 8.89)	(96.4 ± 93.9)	(441 ± 332)	(133 ± 77.7)
Heart	1.07 ± 0.611	2.27 ± 0.611	7.80 ± 4.24	10.0 ± 2.26	7.87 ± 1.15
	(75.3 ± 32.4)	(129 ± 51.0)	(40.4 ± 9.91)	(264 ± 203)	(191 ± 79.3)
Brain	0.0936 ± 0.0187	0.375 ± 0.359	0.370 ± 0.282	0.953 ± 0.507	0.821 ± 0.611
	(6.82 ± 1.29)	(20.1 ± 18.4)	(2.03 ± 1.48)	(22.0 ± 24.3)	(25.9 ± 26.7)
Fat	0.150 ± 0.0803	0.166 ± 0.0335	0.252 ± 0.130	N.D.	N.D.
	(9.59 ± 2.62)	(9.44 ± 3.27)	(1.29 ± 0.410)	(N.C.)	(N.C.)
**4 h**					
Plasma	0.00907 ± 0.00231	0.0348 ± 0.00356	0.115 ± 0.0451	0.106 ± 0.0361	0.0612 ± 0.0283
Liver	0.800 ± 0.0001	3.47 ± 0.462	15.2 ± 7.73	22.1 ± 13.1	17.1 ± 4.60

	(88.2 ± 28.1)	(100 ± 5.94)	(129 ± 24.6)	(194 ± 63.0)	(321 ± 173)
Kidney*	N.D.	3.00 ± 1.41	19.7 ± 5.40	27.7 ± 14.8	25.7 ± 4.81
	(N.C.)	(92.7 ± 49.1)	(180 ± 47.6)	(263 ± 112)	(465 ± 148)
Small intestine	N.D.	91.6 ± 12.1	187 ± 139	375 ± 284	246 ± 127
	(N.C.)	(2651 ± 436)	(1813 ± 1748)	(3991 ± 2865)	(4203 ± 1830)
Large intestine	N.D.	4.40 ± 1.20	241 ± 201	144 ± 102	187 ± 170
	(N.C.)	(125 ± 21.9)	(2405 ± 2588)	(1736 ± 1698)	(2566 ± 1602)
Stomach	N.D.	10.1 ± 1.85	45.0 ± 52.9	68.2 ± 60.2	34.7 ± 15.4
	(N.C.)	(290 ± 34.9)	(288 ± 293)	(917 ± 1035)	(695 ± 432)
Lung	2.13 ± 0.611	3.47 ± 1.29	21.2 ± 23.2	20.6 ± 4.24	12.7 ± 3.45
	(234 ± 16.0)	(103 ± 45.6)	(138 ± 124)	(168 ± 57.6)	(234 ± 112)
Heart	0.533 ± 0.231	2.53 ± 1.01	17.8 ± 13.3	53.6 ± 61.7	9.33 ± 2.84
	(57.3 ± 9.90)	(75.3 ± 36.9)	(126 ± 52.5)	(504 ± 502)	(169 ± 61.3)
Brain+	N.D.	0.147 ± 0.0710	1.21 ± 0.0322	1.67 ± 0.210	0.664 ± 0.237
	(N.C.)	(4.10 ± 2.26)	(10.1 ± 4.21)	(16.7 ± 4.26)	(14.0 ± 10.4)
Fat	0.0718 ± 0.0433	0.0652 ± 0.0173	0.353 ± 0.000849	0.366 ± 0.250	0.387 ± 0.326
	(9.10 ± 5.00)	(1.85 ± 0.337)	(2.93 ± 1.15)	(4.43 ± 4.39)	(10.6 ± 11.4)
**8 h**					
Plasma	N.D.	0.0272 ± 0.0389	0.0710 ± 0.0250	0.0858 ± 0.0490	0.0879 ± 0.0212
Liver	N.D.	3.47 ± 1.80	6.80 ± 0.556	6.27 ± 1.01	6.40 ± 1.70
	(N.C.)	(50.7 ± 17.0)	(120 ± 29.4)	(93.2 ± 54.9)	(66.3 ± 2.29)
Kidney	N.D.	2.80 ± 1.13	19.3 ± 5.52	11.9 ± 2.34	14.1 ± 3.89
	(N.C.)	(32.5 ± 19.9)	(280 ± 49.7)	(196 ± 164)	(166 ± 56.9)
Small intestine	N.D.	87.7 ± 34.1	16.4 ± 10.2	12.9 ± 4.64	59.7 ± 49.3
	(N.C.)	(1627 ± 1357)	(251 ± 148)	(172 ± 59.1)	(706 ± 634)
Large intestine	N.D.	38.5 ± 43.8	45.3 ± 28.6	48.0 ± 29.0	102 ± 127
	(N.C.)	(1028 ± 1549)	(691 ± 477)	(798 ± 779)	(1228 ± 1606)
Stomach	N.D.	9.87 ± 2.41	17.6 ± 9.06	8.53 ± 3.84	18.4 ± 11.7
	(N.C.)	(165 ± 78.4)	(250 ± 110)	(110 ± 34.4)	(227 ± 182)
Lung	1.33 ± 0.611	4.00 ± 0.800	7.07 ± 7.49	10.9 ± 3.63	7.07 ± 4.09
	(N.C.)	(67.4 ± 33.5)	(97.6 ± 102)	(148 ± 54.6)	(82.8 ± 55.1)
Heart	8.00 ± 4.53	2.13 ± 0.833	3.60 ± 0.566	3.07 ± 0.611	3.80 ± 0.283
	(N.C.)	(32.4 ± 8.92)	(60.8 ± 38.6)	(52.7 ± 48.0)	(50.4 ± 7.90)
Brain	N.D.	0.135 ± 0.00446	0.174 ± 0.0274	0.195 ± 0.145	0.402 ± 0.334
	(N.C.)	(2.42 ± 1.59)	(2.68 ± 1.15)	(2.44 ± 1.31)	(5.24 ± 5.03)
Fat	0.104 ± 0.0339	0.341 ± 0.178	N.D.	N.D.	0.192 ± 0.0624
	(N.C.)	(5.63 ± 1.87)	(N.C.)	(N.C.)	(2.21 ± 0.726)
**10 h**					
Plasma**	N.D.	0.0674 ± 0.0282	N.D.	0.0314 ± 0.0290	0.0160 ± 0.00453
Liver	N.D.	2.93 ± 1.29	N.D.	N.D.	N.D.
	(N.C.)	(43.5 ± 3.18)	(N.C.)	(N.C.)	(N.C.)
Kidney	N.D.	3.20 ± 0.0001	4.40 ± 2.26	3.80 ± 3.11	3.20 ± 1.13
	(N.C.)	(42.9 ± 14.7)	(N.C.)	(131 ± 22.0)	(219 ± 133)
Small intestine	N.D.	15.2 ± 11.3	N.D.	7.60 ± 2.83	7.20 ± 2.83
	(N.C.)	(201 ± 49.5)	(N.C.)	(349 ± 232)	(443 ± 51.6)
Large intestine	N.D.	12.5 ± 5.00	8.80 ± 0.566	30.0 ± 18.7	24.0 ± 21.5
	(N.C.)	(205 ± 119)	N.D.	(2144 ± 2574)	(1760 ± 1841)
Stomach	N.D.	5.87 ± 3.26	2.20 ± 0.849	21.0 ± 25.2	3.60 ± 0.566
	(N.C.)	(84.5 ± 12.2)	N.D.	(521 ± 321)	(240 ± 103)

Lung	2.00 ± 0.001 (N.C.)	7.33 ± 1.40 (125 ± 68.2)	5.00 ± 0.849 (N.C.)	10.4 ± 0.001 (577 ± 533)	5.80 ± 1.41 (391 ± 199)
Heart	4.80 ± 2.26 (N.C.)	1.07 ± 0.462 (19.4 ± 15.3)	N.D. (N.C.)	N.D. (N.C.)	N.D. (N.C.)
Brain	N.D. (N.C.)	N.D. (N.C.)	N.D. (N.C.)	0.126 ± 0.0325 (6.17 ± 4.66)	0.132 ± 0.0359 (8.94 ± 4.77)
Fat	0.0871 ± 0.0139 (N.C.)	N.D. (N.C.)	N.D. (N.C.)	N.D. (N.C.)	N.D. (N.C.)

*MBR-NDA and MBR-PG mice were significantly different ($p < 0.05$) from MBR mice.
**MBR-EFA and MBR-PG mice were significantly different ($p < 0.05$) from MBR mice.
+MBR-NDA mice was significantly different ($p < 0.05$) from MBR mice.
